# Modelling potential distribution of the invasive box tree moth across Asia, Europe, and North America

**DOI:** 10.1371/journal.pone.0302259

**Published:** 2024-04-26

**Authors:** M. Lukas Seehausen, Alex Rimmer, Abigail Wiesner, Marc Kenis, Cynthia Scott-Dupree, Sandy M. Smith

**Affiliations:** 1 CABI, Risk Analysis & Invasion Ecology, Delémont, Switzerland; 2 Institute of Forestry and Conservation, University of Toronto, Ontario, Canada; 3 School of Environmental Sciences, University of Guelph, Guelph, ON, Canada; University of Thessaly School of Agricultural Sciences, GREECE

## Abstract

The box tree moth *Cydalima perspectalis* (Walker) (Lepidoptera: Crambidae) (BTM) is a native moth throughout eastern Asia, having recently become invasive in Europe (2007) where it feeds on boxwood (= box tree), *Buxus* spp. The moth rapidly spread across Europe and the Caucasus causing damage to both ornamental and wild *Buxus*. In 2018, *C*. *perspectalis* was found in Toronto, ON, Canada, and has since spread south into the US. To better predict where the moth will establish and have significant impact on ornamental trade in North America, we used most recent scientific literature and distribution points to update the temperature and diapause indices of an existing ecoclimatic CLIMEX model. The model parameters provided a good fit for the potential distribution of BTM compared to its known distribution across eastern Asia and in Europe. Interestingly, our results suggest that the current native distribution in Asia is incomplete and that further expansion is also possible in its introduced range, especially in northern Europe, along the Mediterranean coast of Africa, and eastward to central Russia. In North America, the model predicts that most of North America should be climatically suitable for the moth’s establishment, with the exception of Alaska and the northern territories of Canada, as well as higher elevations in the Rocky Mountains and southern hot and dry areas. Our study highlights the importance of the CLIMEX model to assess the risk of BTM spreading in its newly invaded areas, especially North America, and its use to help make decisions in terms of regulatory dispersal restrictions and choice of management options.

## Introduction

Human-mediated transport of non-native species to new regions has increased rapidly as a result of global trade and the rising number of transported species that have been able to establish successfully and become invasive in their new location [1, 2]. Insects now make up a significant portion of all invasive species worldwide and there is no sign that this trend will slow [3, 4]. Despite the invasion process being complex and reliant on a combination of abiotic (*e*.*g*., temperature and soil moisture) and biotic (*e*.*g*., host quality, voltinism, and natural predators) factors [5–7], the establishment of invasive species suggests that they have the ability to reproduce and spread rapidly in their invaded ranges, leading to large negative ecological, societal, and economic consequences [8, 9].

The box tree moth (BTM), *Cydalima perspectalis* (Walker) (Lepidoptera: Crambidae), is a lepidopteran native to eastern Asia including China and Taiwan [10], Japan [11], South Korea [12], India [13], Pakistan [14], and Far East Russia [15]. It feeds on at least 10 species of *Buxus* (Buxaceae) trees and shrubs in eastern and south-eastern Asia [16], all commonly referred to as ‘box tree’ or ‘boxwood’. The moth occurs in natural box tree stands and commercial plantations, however in Asia it is described as a pest of ornamental *Buxus* spp. commonly planted in public parks and private gardens.

In Europe and North Africa (Morocco and Algeria), *Buxus sempervirens* (= *B*. *colchica*) and *B*. *balearica* occur naturally as wild plants and their distribution extends to the east as far as Turkey, Georgia, Iran, and Kazakhstan [17]. In central and southern Europe, *Buxus* spp. are commonly found growing in the understory of broadleaf forests [17], while in the Caucasus they grow in the canopy as tall dominant trees [18, 19]. Additionally, *Buxus* spp. in Europe are common ornamental plants, often found in palace and private gardens, and public parks where they are preferred by gardeners because they can be easily trimmed into complex shapes and grow slowly with minimal care.

BTM was first detected in Europe in southwestern Germany and the Netherlands during 2007 [20, 21]. It spread rapidly across Europe and western Asia [22] and was recently found in North Africa [23]. Damage to *Buxus* spp. is mainly caused by larvae feeding on foliage, however, in cases of complete defoliation, the bark of box trees can also be removed [24]. Repeated defoliation leads to plant death after only two years [24], although some trees can partially recover by growing small branches at the base over several years. In parks and gardens, the defoliation is mainly seen as a nuisance and insecticides can be applied [25, 26]. Insecticides are not usually allowed for control in natural stands and this leads to ecological and societal consequences beyond simple aesthetic damage [24, 27]. The gradual disappearance of boxwood in natural stands has consequences for biodiversity, as a multitute of species are dependent on it and therefore are at risk of becoming extinct. In addition, important ecosystem functions, such as soil stability and water quality, may degrade in natural stands and certain cultural practices become lost as boxwood dies out [27].

The first discovery of BTM in North America was in 2018 at three residential locations in Toronto, Canada [28]. While boxwood does not occur naturally in Canada or the US, it is a common plant in private and public gardens and this has likely facilitated rapid spread of BTM following its North American introduction [29]. In 2021, BTM was detected by the Canadian Food Inspection Agency (CFIA) at a nursery facility in Canada outside the known Toronto infestation (about 20 km from the US border) and similarly at a nursery facility in the US by the USDA-APHIS [30]. In May of the same year, the US banned importation of plants-for-planting in the genera *Buxus* spp., *Euonymus* spp., and *Ilex* spp. from all Canadian provinces, which has resulted in significant economic trade conflict between Canada and the US due to the high value of these ornamentals in both countries. BTM has continued to spread on both sides of the border; as of the end of 2023, it has been found as far south as Cincinnati and Cape Cod, US, and as far north as Ottawa and Montréal, Canada (see Results for more information).

In Europe, BTM is known to have a comparatively high rate of spread that clearly exceeds its biological flight capacity [31] suggesting a human-mediated pathway. Because boxwood is commonly traded between countries, and even continents [21, 32], BTM can be moved long distances through the commercial transport of nursery plants. As seen in Europe, once moved to a new location, the moth has a high likelihood of establishment due to combined factors of widespread boxwood populations occurring naturally in the wild or as ornamental plantings, and to a lack of specialized natural enemies [16, 22, 31]. Thus, it appears that abiotic factors are the main drivers restricting natural BTM dispersal, of which temperature, humidity, and daylength are likely the most important, and this makes it a suitable candidate for ecoclimatic niche modeling to predict its potential natural distribution and spread in new geographic regions [33]. More specifically, BTM may not be able to establish in colder climates (*e*.*g*., extreme northern or southern latitudes or high altitudes) due to mortality under cold winter temperatures during diapause or insufficiently high temperatures preventing completion of one full generation. Similarly, it may fail to establish in hotter climates (*e*.*g*., equatorial and arid regions) due to heat stress and dessication. Finally, population growth may also be impacted by short daylengths that induce diapause (see the section Fitting CLIMEX parameters for more information).

To our knowledge, two models have been developed to predict potential distribution of BTM for Europe, one CLIMEX [34] and one MaxEnt model [35]. While MaxEnt models are based on the known distribution of organisms and their surrounding environment [36], CLIMEX models add information on the species’ biology including temperature-dependent development, sensitivity to humidity, and changes in daylength [33]. Here, we include most recent knowledge about BTM biology and distribution in North America to update the existing CLIMEX model and improve the prediction for its global potential distribution based on climatic niche modelling.

## Material and methods

### Occurence records

The current occurence of the BTM was gathered through a combination of records listed in the Global Biodiversity Information Facility (GBIF) database, a literature search, and pheromone trapping records (the latter available only for Ontario, Canada since 2019). The GBIF entries for BTM [37] were first reduced to those with available coordinates and then the points were visualized in the Quantum Geographic Information System (QGIS) to identify erroneous records. When identification records of obvious outlier points were verified, they were removed if misidentified or if a record was not verified by a photo, specimen or DNA information.

Additional distribution records were collected through a thorough search of the literature using Google Scholar and CAB Abstracts, including scientific and specialized articles, reports, and presentation abstracts. From here, provided coordinates were noted or center coordinates of provided locations (at least at the provincial level) were searched on Google Maps. Finally, for a more detailed distribution in Ontario, Canada, data from pheromone traps were included (detailed methods on pheromone trapping in Wiesner *et al*. [29]). All records assembled for the present study can be found in S1 File. The Saga thinning tool was used in QGIS to reduce the point density and visualize the extent of distribution in Asia and Europe without compromising the visibility of the model results. Points with slightly inaccurate coordinates, *e*.*g*., placed in water just off a coast, were manually corrected by moving them to the closest land mass.

### Model description

The process of bioclimatic modelling using CLIMEX 4 here was based on detailed work by Kriticos *et al*. [33]. CLIMEX models describe a species’ response to climatic variables, which define its relative performance in a given location as well as the limit of its geographical distribution. Several indices can be used to describe the potential growth and survival of a species’ population and these can be broadly grouped into growth and stress-related indices. Population growth is defined by the Weekly Growth Index (*GI*_*W*_) that combines the response to weekly temperature (*TI*), moisture (*MI*), and diapause indices (*DI*) and then averaged to calculate an Annual Growth Index (*GI*_*A*_). Stress indices limit a species’ persistence in a given area based on weekly cold (*CS*), heat (*HS*), dry (*DS*), and wet stress (*WS*), all which accumulate at a defined rate. All stress indices are then combined into an Annual Stress Index (*SI*_*A*_).

A species’ distribution can also be limited if (1) thermal conditions do not allow for sufficient heat accumulation during one season for a species to complete at least one generation, and (2) conditions cannot be met for a species with obligate diapause to complete diapause development. An overall annual index of climatic suitability, the Ecoclimatic Index (*EI*), integrates *GI*_*A*_, *SI*_*A*_, and the limiting conditions to rank them between EI = 0 for locations where the species is not able to persist and to EI = 100 for locations that are optimal for the species. However, it is important to note that EI values close to 100 are only achievable under constant ideal conditions, such as in incubators or for some species in tropical regions. Generally, values >30 represent very favourable conditions [33] and it has been shown that lower values are often sufficient to support substantial population densities (*e*.*g*., [38]).

The 30′-gridded CliMond historical data set (1995H_V2) was used to approximate the current climate [39] where data are based on CRU CL2.0 and WorldClim spanning 30 years centered on the year 1995. One of the underlying assumptions of CLIMEX is that a species’ known distribution infers the climatic conditions it can tolerate. Therefore, the known species distribution can be used for model calibration in cases where some parameters are unknown (*e*.*g*., the known distribution in the area of origin) or for model validation if the species occurs in additional locations that were not used for model calibration (*e*.*g*., in an already invaded continent).

### Fitting CLIMEX parameters

The starting parameters for the model were taken from Nacambo *et al*. [34] and updated with recent information about the environmental factors affecting the performance of BTM. All values of the parameters used in the present model and those used by Nacambo *et al*. [34] are listed in Table 1.

**Table 1:** Values for parameter settings used in the CLIMEX model to calculate the potential geographic distribution of box tree moth (BTM) *Cydalima perspectalis*.

Group	Parameter	Description	Nacambo *et al*. 2014	Present study
Moisture index	SMO	Limiting low soil moisture	0.01	0.01
	SM1	Lower optimal soil moisture	0.1	0.20
	SM2	Upper optimal soil moisture	1.5	1.50
	SM3	Limiting high soil moisture	2.5	2.50
Temperature index	DV0	Limiting low temperature (°C)	9.5	8.4
	DV1	Lower optimal temperature (°C)	15.0	26.0
	DV2	Upper optimal temperature (°C)	27.5	31.0
	DV3	Limiting high temperature (°C)	35.0	35.0
Diapause index	DPD0	Induction day length	13.5	13.5
	DPT0	Induction temperature (°C)	20	25.0
	DPT1	Termination temperature (°C)	0	0
	DPD	Development days	45	45.0
	DPSW	Indicator for winter diapause	0	0
Cold stress	TTCS	Temperature threshold (°C)	-20	-20
	THCS	Temperature rate	-0.001	-0.001
Heat stress	TTHS	Temperature threshold (°C)	40	32
	THHS	Temperature rate	0.005	0.005
Day-degree accumulation above DV0	DV0	Limiting low temperature (°C)	9.5	8.4
	DV3	Limiting high temperature (°C)	35.0	35.0
	MTS	Model time step	7	7
Day-degree accumulation above DVCS	DVCS	Cold stress DD threshold temperature (°C)	8	8
	DV4	Dummy parameter	100	100
	MTS	Model time step	7	7
Day-degree accumulation above DVHS	DVHS	Heat stress DD threshold temperature (°C)	31	31
	DV4	Dummy parameter	100	100
	MTS	Model time step	7	7
Degree-days per generation	PDD	Minimum degree-days above DV0 to complete one generation	540	665

### Moisture index

Since BTM has no life stages in the soil, the Moisture Index, which mainly applies to soil conditions, will only affect BTM indirectly through its host plant, although it can also be assumed that soil moisture is some indication of aerial moisture surrounding the plant. Parameters used to calculate the Moisture Index were the same as in the model from Nacambo *et al*. [34] except for lower optimal soil moisture, which was raised by 0.1. As such, the limiting low soil moisture of 0.01 (soil moisture content at 1% of its holding capacity) and the limiting high soil moisture of 2.5 (water content 150% higher than the soil holding capacity meaning that there is heavy water run-off) indicate that plant and insect growth are not highly dependent on extreme moisture levels. This is in accordance with the occurrence of the moth and its host plant in winter-dry areas in central and north-east China [34]. However, optimal moisture conditions are assumed to be between 20 and 150% of the soil holding capacity, which are moisture indices of the CLIMEX temperate climate template. Irrigation of plants in cities is not considered here, which may explain some outliers in the observed original distribution, such as in Lhasa (Tibet) and Xining (Qinghai), China (S1 File).

### Temperature index

**Fig 1:**
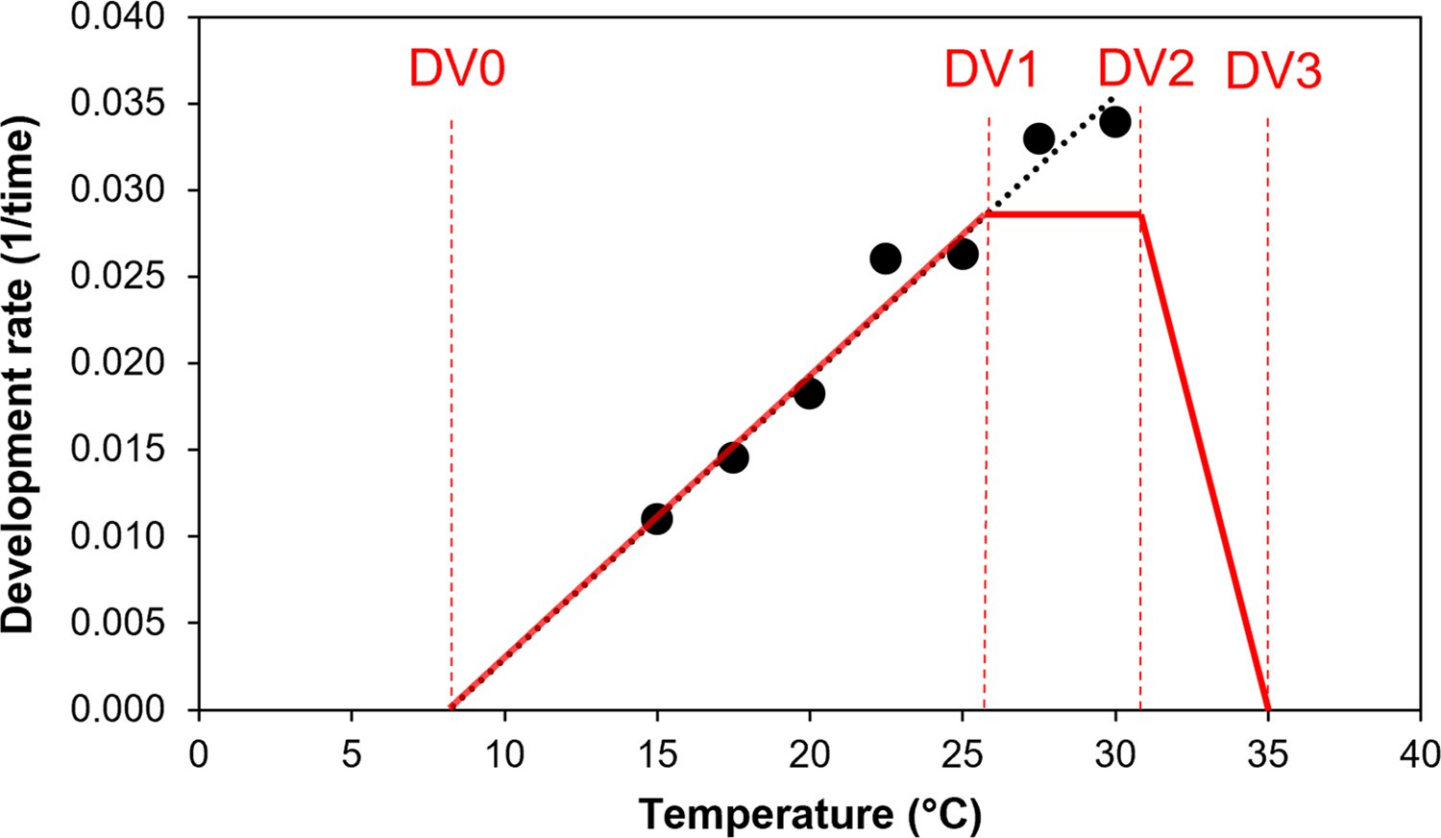
Mean overall developmental rates (1/developmental time in days) from egg to adult for box tree moth (BTM), *Cydalima perspectalis*, at temperatures between 15 and 30°C and corresponding regression (black line with equation). The red lines indicate the simplified non-linear developmental rate curve used to determine the model parameters for the temperature index.

Values for the Temperature Index are based on temperature-dependent developmental rates from the literature and our own observations. Most developmental rates in the literature are stage-specific, *i*.*e*., separate calculations for eggs, larvae, and pupae (*e*.*g*., [34, 40]). However, for the Temperature Index of CLIMEX, values from the developmental rates of an entire subadult generation are needed. Therefore, we used the data from Nacambo *et al*. [34] to calculate the linear regression parameter estimates (Table 1) and the overall developmental times of BTM from egg to adult at seven temperatures between 15 and 30°C. Overall developmental times at each temperature were determined by calculating the mean of individual developmental times at each developmental stage and then adding them together. The resulting points were displayed graphically as developmental rates (1/developmental time in days) and then used to estimate the index parameters based on the simplified calculations of a developmental rate curve used by CLIMEX ([33]; Fig 1). The limiting low temperature (DV0) we used in the model (= 8.4°C) was the x-intercept of the developmental rate regression line (*i*.*e*., the lower temperature threshold at which no more development occurs). The range between the optimal lower (DV1) and upper (DV2) temperature should represent the range over which the species grows at near optimal rates (*i*.*e*., ~ 90–100% of the maximum) and is usually ~ 4°C [33].

To our knowledge, there are no data available on the optimum temperature for the fastest development or upper temperature threshold for development of *C*. *perspectalis* because it has only been reared at temperatures where overall developmental rates are still fairly linear (< = 30°C) (see [34, 40]). However, stage-specific developmental rates from Swiss populations of the moth show a reduced increase in developmental rate at 30°C compared to lower temperatures, especially for larvae [34]. Therefore, we assumed that the fastest development would occur close to 30°C and consequently we set DV1 = 26 and DV2 = 31°C. In accordance with Nacambo *et al*. [34], the limiting high temperature at which development ceases (DV3) was estimated to be 35°C. For the weekly (MTS = 7) degree-day accumulation above DV0, the lower and upper threshold temperatures were set to DV0 and DV3, respectively. The degree-days per generation (PDD = 665) were calculated as the reciprocal of the regression line’s slope (= 625), which is the sum of degree-days required for 50% of the individuals to complete development to adulthood, plus 40 degree-days that are approximately needed for the pre-ovipositional period of females (38.5 degree-days above 8.1°C, [41]).

### Diapause index

BTM is known to undergo a winter diapause (therefore DPSW = 0), which is induced in the early larval instars by short daylengths. The sensitive developmental stages for the photoperiodic induction of diapause have been found to be between the 1^st^-and 3^rd^-larval instar [11, 41]. In most regions of China, larvae cease their development and build overwintering cocoons in 2^nd^- to 4^th^-larval instars, however, in some they may not do so until the 5^th^-larval instar or even as mature larvae (reviewed by [16]). Maruyama & Shinkaji [42] found that diapause occurred in the 4^th^—and 5^th^-larval instars in Japan while in Europe, studies report that BTM larvae enter diapause mainly in 3rd- and 4^th^- larval instars [34, 43], with a few individuals in north-central France doing so in the 5^th^-larval instar [44].

Values for the diapause index parameters determined by Nacambo *et al*. [34] were generally used in the model, except for the diapause induction temperature. Short daylengths have been found to be the main factor inducing diapause in BTM; the critical daylength at which 50% of individuals go into diapause [11] being 13.5 h at temperatures up to 25°C [34, 41, 43]. Hence, diapause induction daylength (DPD0) was set at 13.5 h although the induction temperature parameter value (DPT0) was raised from 20 to 25°C.

Factors influencing diapause termination in *C*. *perspectalis* have only recently been studied in detail. Poitou *et al*. [44] found that temperature, not photoperiod, was the main factor determining diapause termination. Similarly, Nacambo *et al*. [34] showed that the moth required one and a half to two months of cold exposure to terminate diapause in Swiss populations, although spontaneous termination of diapause has also been recorded after about 100 days without cold exposure, *i*.*e*., for a BTM population in Spain’s eastern Pyrenees [43]. To approximate these characters in the model, diapause termination temperature (DPT1) was left at zero to indicate that termination is temperature-independent, and the minimum number of developmental days below DPT0 (DPD) was set at 45 to reflect values in Nacambo *et al*. [34]. Systematically changing parameter values for induction temperature and the minimum number of days below this temperature to better reflect southern climates had little effect on the predicted distribution and only slightly changed EI values.

### Stress parameters

The parameter values for cold stress or cold hardiness of overwintering larvae were left at those suggested by Nacambo *et al*. [34], with the rate of stress accumulation THCS = -0.001 starting at TTCS = -20°C based on the moth’s known distribution in northern China and Russian Far East where temperatures commonly reach -30°C. For the weekly (MTS = 7) calculation of degree-day-based cold stress, the temperature threshold was set at DVCS = 8°C, which corresponds to the rounded limiting low temperature for development of the temperature index. The temperature threshold for heat stress was adjusted here to TTHS = 32°C and an accumulation rate of THHS = 0.007. Thus, heat stress accumulates starting at 1°C above the upper optimal temperature (DV2) at a rate that reflects the decreasing slope to 35°C, the limiting high temperature (DV3) at which no population growth can take place.

Wet and dry stress indices were not used here as the native distribution of the BTM in Asia seems to be minimally or not at all affected by these factors. The temperature threshold for the weekly calculation of the degree-day-based heat stress was set to 31°C from which it is assumed there will be decreased development based on the upper optimal temperature defined for the temperature index.

## Results

### Predicted and observed distribution and phenology in east and south-east Asia

**Fig 2:**
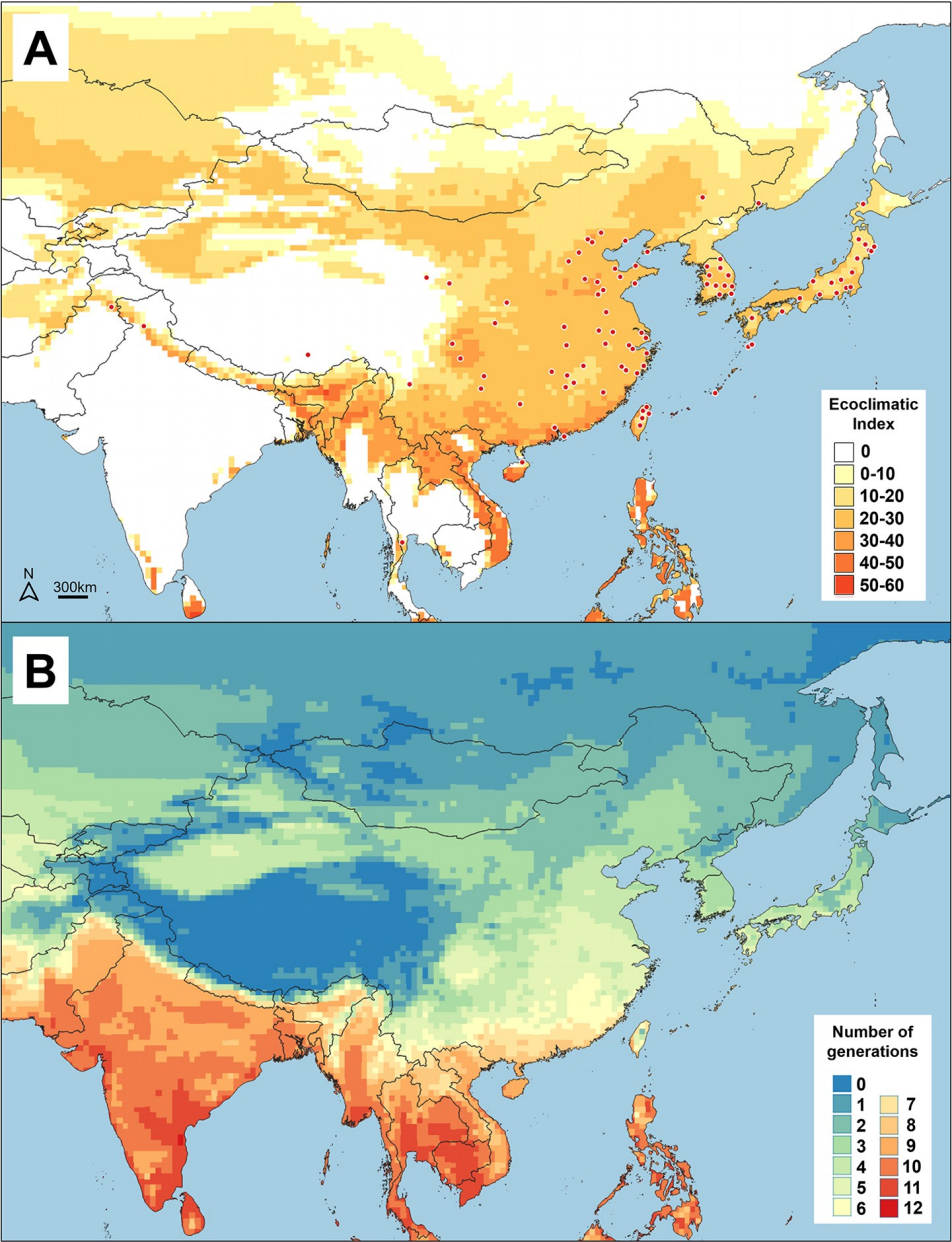
(A) Potential (yellow to red areas) and observed (red points) native distribution and (B) predicted number of generations of box tree moth (BTM), *Cydalima perspectalis*, in south-eastern Asia. The maps were created using CLIMEX and QGIS software.

The predicted distribution of BTM in Asia matched the observed distribution with one exception, Lhasa, Tibet (Fig 2A). In northern latitudes, the predicted distribution is likely limited by cold stress and in the northwest and west (Tibet and Mongolia) by a combination of cold stress and an insufficient number of degree-days to complete one full generation. Heat stress accurately predicted the moth’s exclusion from central India. Finally, we did not see restriction of the moth to high altitudes in the south as predicted by Nacambo *et al*. [34], likely due to the fact we used a higher diapause induction temperature (DPT0 = 25°C).

**Fig 3:**
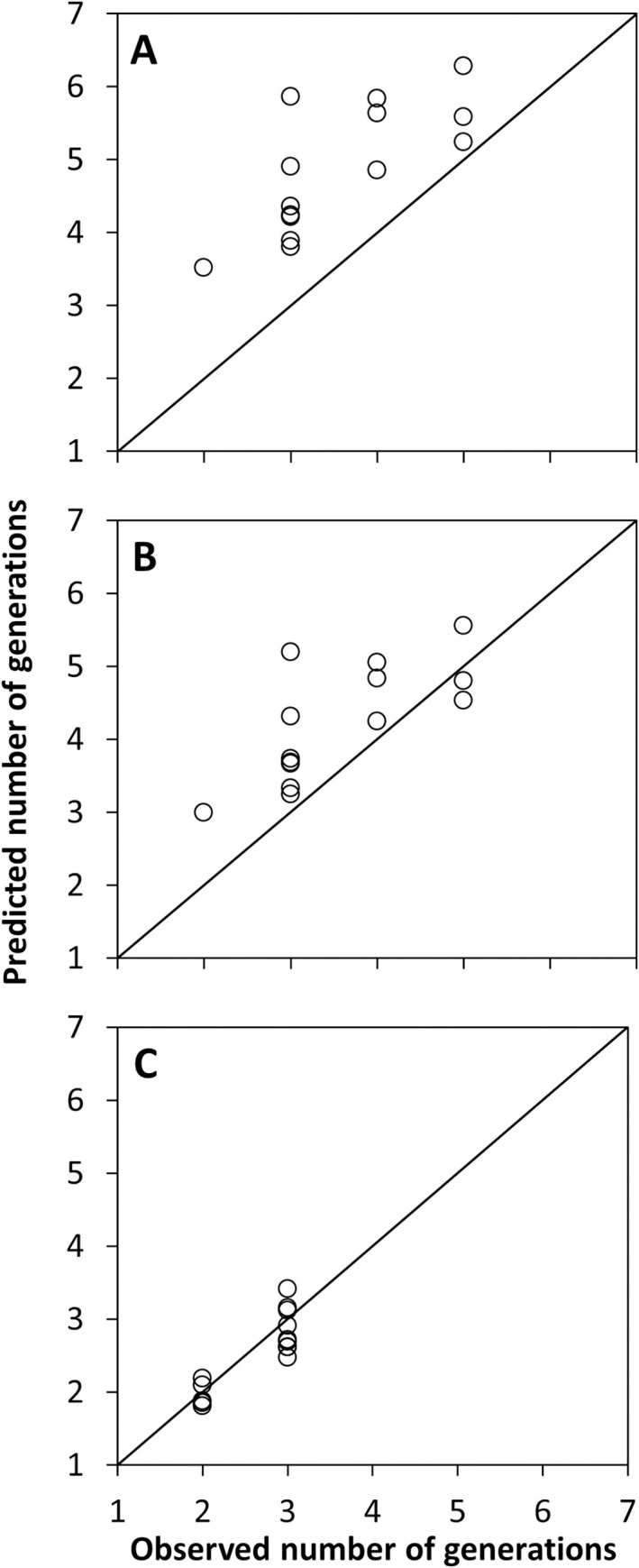
Relationship between predicted and observed number of generations of box tree moth (BTM), *Cydalima perspectalis*, for (A) Asia using Temperature Index (TI) values based on data from a BTM population from Switzerland, (B) Asia using TI values from a Japanese BTM population, and (C) Europe using TI values from a Swiss BTM population. The diagonal line in each panel represents a perfect fit between observed and predicted number of generations.

Our model predicted the number of BTM generations in Asia (Fig 2B) although it was approximately 1.5 times greater, on average, than the number modeled by Wan *et al*. [16] (Fig 3A). This overestimation in number of generations was reduced to between zero (at five observed generations) and one (at two observed generations) when the parameters were calculated similarly but with developmental times [40] and diapause induction daylength [11] from a BTM population in Tokyo, Japan (Fig 3B). The greater accuracy of our model was mainly due to a higher limiting low temperature (calculated here at 11.1°C) and a slightly lower number of degree-days per generation (PDD = 595) that led to later developmental onset in the spring.

### Predicted and observed distribution and phenology in Europe, Western Asia, and North Africa

**Fig 4:**
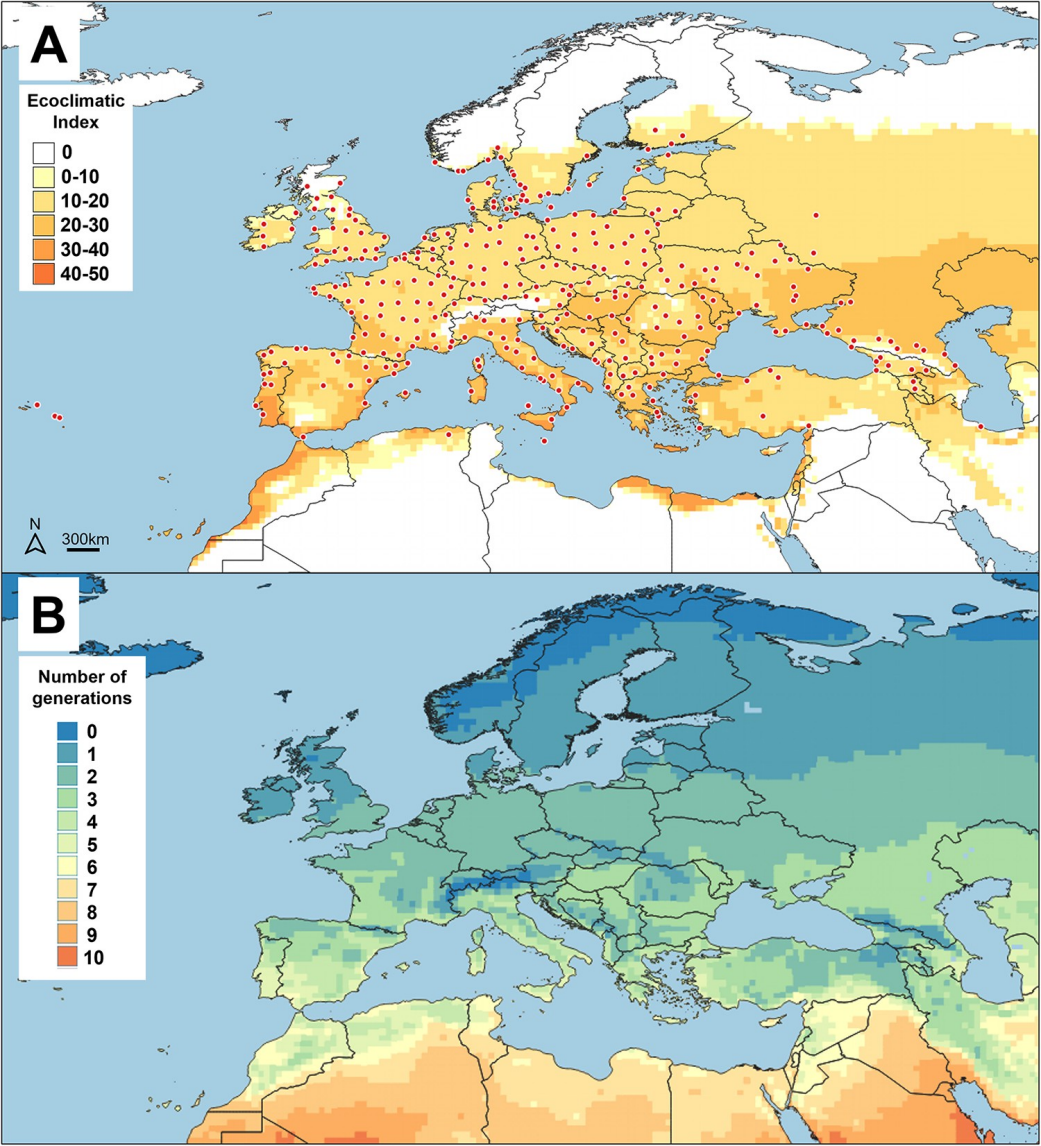
(A) Potential (yellow to orange areas) and observed (red points) invasive distribution and (B) predicted number of generations of box tree moth (BTM), *Cydalima perspectalis*, in Europe, western Asia, and northern Africa. The maps were created using CLIMEX and QGIS software.

The predicted distribution of BTM is a close match to its observed distribution in Europe (Fig 4A) where it is present at northern limits in Scotland [45] and Sweden [46], and more recently also in Norway [47, 48]. To the south, BTM has reached the African continent in Algeria [23], southern Europe in Sicily [49] and Malta [50], the Greek Peloponnese [51], and most recently the province Hatay in southern Turkey [52]. Results from our model suggest that BTM has the potential to spread further along the Mediterranean coast of Africa, crossing from Gibraltar [53] into Morocco, and also into the southern coast of the Black and Caspian Seas. To the west, BTM is well established in Portugal and even on the Azores [54], but to the east the model suggests that there is still potential for expansion, especially into central Russia.

In European countries where the number of BTM generations has been documented, two or three generations have been most commonly reported (see S1 File). Our model accurately predicts this number of observed generations here (Fig 3C), however, because information is lacking from southern Europe where BTM may undergo >3 generations, the accuracy of the predictions for such regions remains unknown (Fig 4B).

### Predicted distribution and phenology in North America

**Fig 5:**
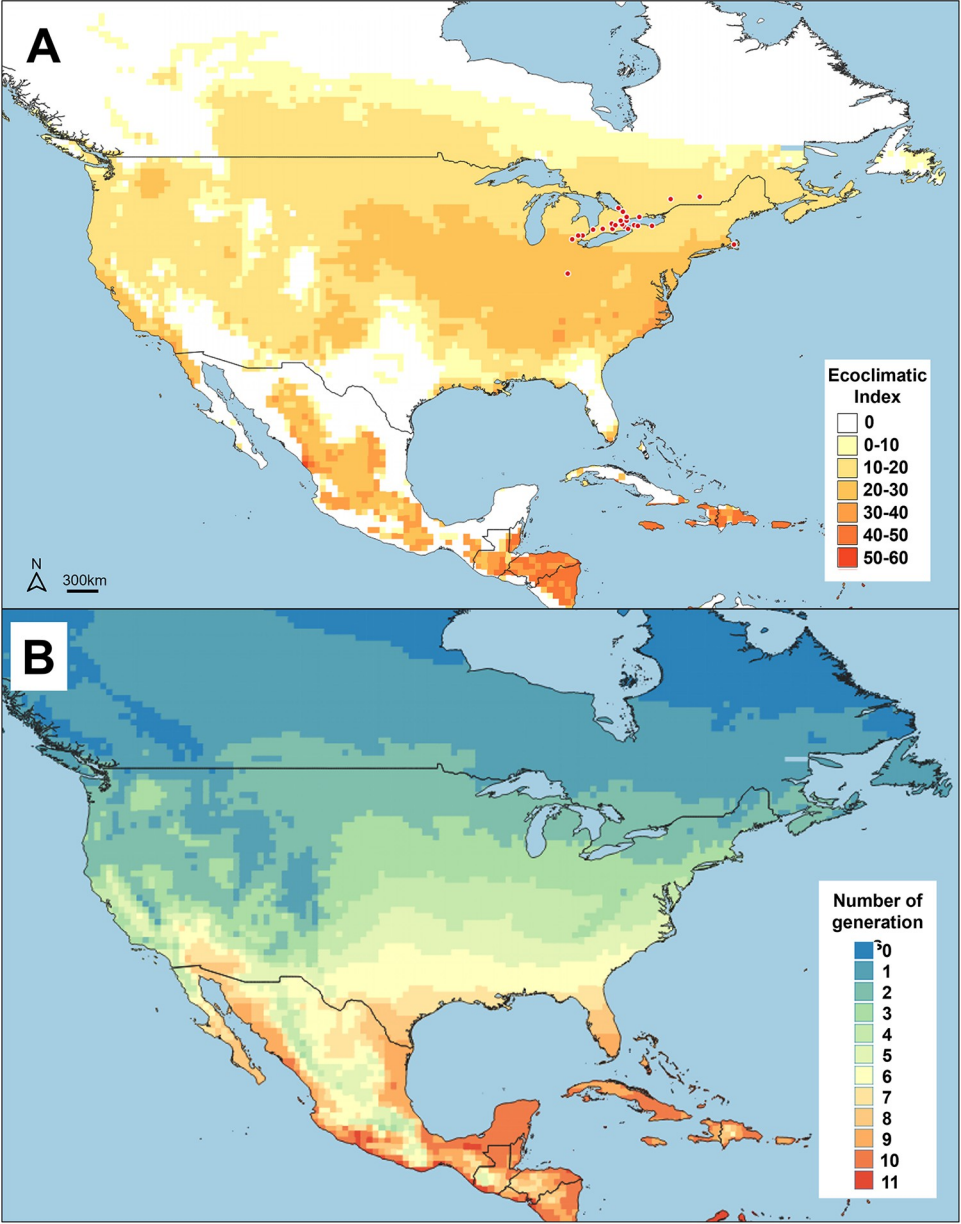
(A) Potential (yellow to red areas) and observed (red points) invasive distribution and (B) predicted number of generations of box tree moth (BTM), *Cydalima perspectalis*, in North America. The maps were created using CLIMEX and QGIS software.

**Fig 6:**
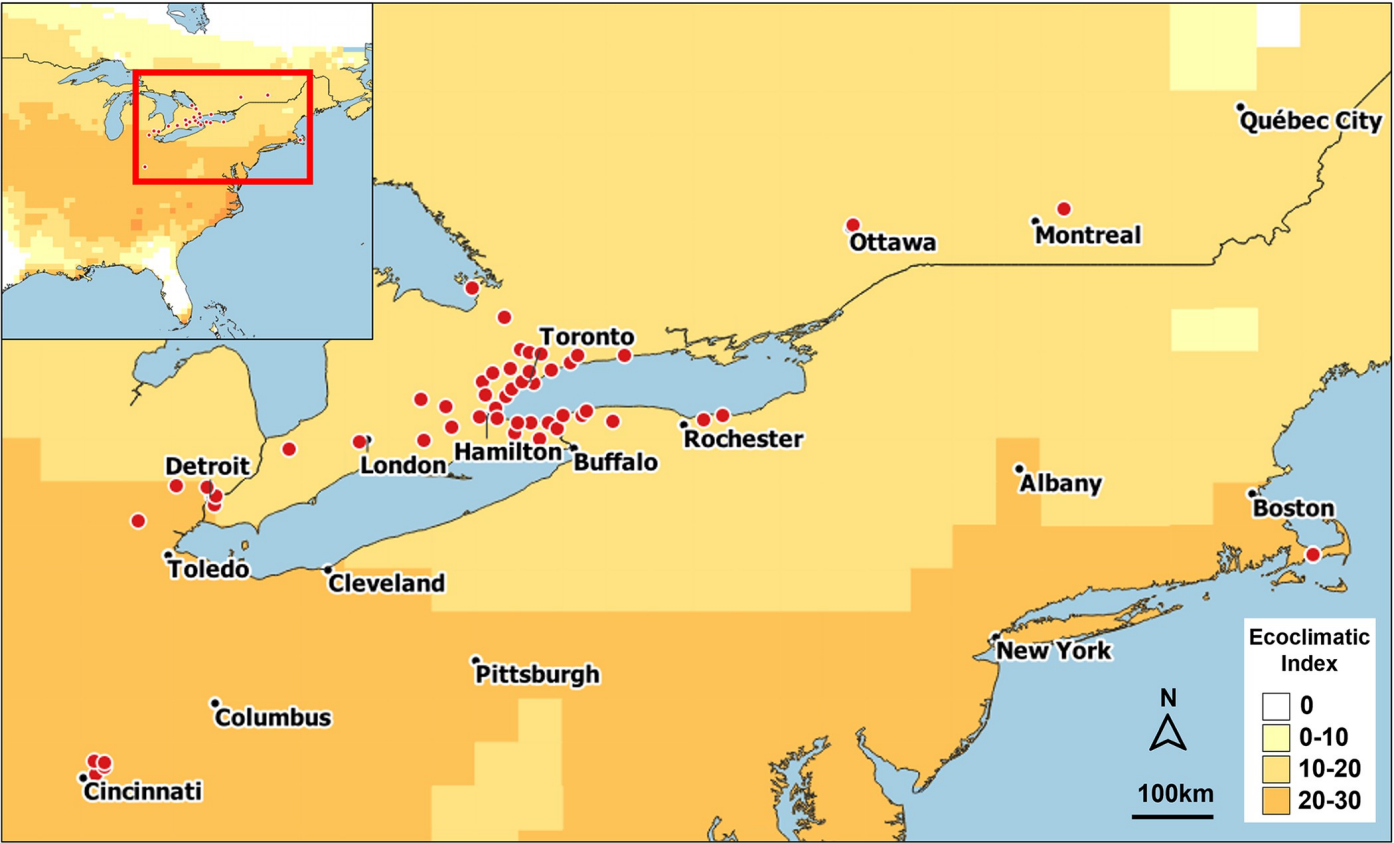
Observed distribution of box tree moth (BTM), *Cydalima perspectalis*, Canada and the US (red points) reported until the end of 2023 through pheromone traps and verified GBIF records. Colours in the maps correspond to values of the Ecoclimatic Index in Fig 5A. The maps were created using CLIMEX and QGIS software.

In North America, the model predicted a climatically suitable area for BTM in the north from Edmonton, Canada through to the US and southcentral Mexico, as well as from the Pacific to the Atlantic coast in the US, excluding most of Florida (Fig 5A). The Rocky Mountains and other mountain ranges in western Canada and the US appear to be generally unsuitable for BTM development because the necessary degree-days are not available to complete a full generation (Fig 5B). Additionally, the semi-arid and desert regions of western US and Mexico are largely unsuitable because the dry summers restrict development of both BTM and its host plant. On the other hand, the western coastal climates are suitable, at least from the northern part of Mexico and Califonia’s Baja region up north to Vancouver, Canada. In western Canada, the southern parts of the central provinces, Alberta, Saskatchewan, and Manitoba, are all predicted to be suitable for BTM development, as is also eastern Canada including the southern parts of Ontario and Quebec (to the Gaspé Peninsula), all of New Brunswick, Nova Scotia, Prince Edward Island, and parts of Newfoundland. Heat stress is predicted to exclude the moth from certain regions in the southern US and coastal areas of Mexico, as well as most of Florida and the Caribbean Islands. Finally, our model predicts that the climate is very suitable for BTM establishment in central North America between Montréal, Canada, and along Lake Ontario and Lake Erie to Cincinnati in the south where it has recently been discovered (where the EI is between 16 and 26) (Fig 6).

Pheromone trap data of adult BTM collected from Toronto, Canada confirmed the model’s prediction that the moth could complete 2.1 to 2.3 generations per year [29]. A maximum of one generation per year was predicted for BTM in regions north of the Great Lakes (Fig 5B) while the mountainous terrain in British Columbia and the western US suggests patchy suitability. In central and eastern US, the model predicts the number of BTM generations to increase inversely with latitude to a maximum of nine generations in southern Florida and up to 12 in Mexico. Finally, BTM distribution is predicted to be unlikely in southern and southwestern US and Mexico because it would be restricted by heat stress even though these areas could theoretically have the highest number of generations.

See S1 Fig for a world map showing the model results from the Ecoclimtic Index.

## Discussion

The updated CLIMEX model described here for BTM accurately predicts the moth’s potential distribution in its native range across east and south-east Asia, as well as in the invaded ranges of Europe, western Asia, and northern Africa. As such, we used it to project the potential distribution of BTM in its newly invaded region of North America. In so doing, it has provided important new information for risk assessment to help make regulatory decisions in terms of dispersal restrictions and choice of management options.

The Ecoclimatic Index (EI) of the model correctly described the known distribution of BTM in Asia with one exception, Lhasa, Tibet, which may have resulted from either misidentification or more likely due to some unique favourable condition for BTM on ornamental *Buxus* spp. allowing it to establish temporarily. Generally, BTM occurred in a few locations where the model predicted it would not be suitable, however it is possible that favourable conditions, such as watering or warmer microclimates in cities were created in these areas due to human activity. Nacambo *et al*. [34] noted that distributional data from sub-tropical and tropical areas of south and south-east Asia where several *Buxus* spp. are known to occur naturally [55, 56] are missing. The recent observation of BTM in Kaeng Krachan, Thailand (250 m elevation; species ID validated by photo proof on observation.org, 2017) at least partially validates the accuracy of our model to predict the potential distribution of BTM in tropical climates, and to date, is the first record of its unknown distribution in south-east Asia. More evidence is needed to accurately determine the wider distribution of BTM in south and south-east Asia.

Our model also accurately predicted the current observed distribution of BTM in Europe, western Asia, and northern Africa. The moth is now present in all natural *Buxus* spp. stands in Europe, the Caucasus region, and western Asia [24], and has spread well beyond these areas to attack ornamental *Buxus* spp. plants in parks and private gardens. Recent distribution locations added at the northern, southern, and eastern invasion fronts indicate that BTM is continuing to expand, as is suggeted by the model, especially in southern Norway, Sweden, and Finland, along the Mediterranean coast of Africa, and eastward to central Russia. The isolated occurrence of BTM documented in Chelyabinsk, Russia (iNaturalist entry with only photo proof) shows that further spread to the east is possible, although definitive evidence for this location (*e*.*g*., DNA-based identification) and further information, such as winter survival of the moth in this area, are still missing.

Box tree moth has only recently been introduced into North America [28, 29] and therefore its distribution on this continent is so far limited. However, with the exception of Alaska and the northern territories of Canada, the model predicts most of North America will be suitable for the moth’s establishment. On the US west coast, BTM establishment is predicted to be restricted by the dry summer weather, possibly due to unsuitable conditions for its host plant. However, irrigation in private gardens and public parks may provide enough water for the growth of *Buxus spp*. and therefore create a suitable habitat for BTM. The colder climate in the southern regions of western Canada (*e*.*g*., Manitoba, Saskatchewan, Alberta, and British Columbia) will only support one generation per year of the moth, thus its impact on *Buxus* in these regions is likely to be limited. While Europe provides an excellent example as to how fast BTM can invade new areas, it is important to note that apart from Mexico, *Buxus* is not naturally distributed in North America [57] and is present only as an ornamental plant in parks and private gardens. This may slow the rate of natural dispersal in less densely populated areas where *Buxus* spp. are uncommon, although human-mediated spread (*e*.*g*., through commercial movement) is likely to continue to contribute to its accelerated movement between cities and at even longer distances as recorded in horticultural retail facilities across Michigan, Connecticut, and South Carolina in the US [30]. Such predicted spread places many of the native *Buxus* spp. found throughout the Caribbean [57] at risk, even though heat stress may prevent the establishment of BTM in some areas.

While our model accurately describes the known native and invasive distribution of BTM, there are inconsistencies in the predicted number of generations per year between Asia and Europe. The model projects two to three generations per year in Europe and two generations in Ontario, Canada, both supported by observed BTM phenology [29, 34]. Unfortunately, the model does not agree with the observed phenology for BTM in the Hyrcanian Forests, Iran, where it predicts 3–4 generations per year but only two full generations are reported [58]. It remains unclear whether this mismatch is due to the difficulty of accurately identifying overlapping generations or is a problem associated with the model itself. So far, no phenology data are available from other invaded southern countries such as Greece [51] to validate the model predictions in warmer climates. For Asia, the model overestimates the number of annual generations, which is likely because data to define the temperature and diapause index parameters are based on only one European BTM population. When using data from a Japanese population [40] for the index parameters, the predicted number of generations matches the ones observed in Asia much better (Fig 3B). The known variability in these two life-history traits, namely temperature-dependent developmental rates and critical daylength for diapause induction, seems to be responsible for this increased accuracy in predicting the moth’s phenology in Asia. However, limitations in the modeling process within CLIMEX may also impact the accuracy of the predicted number of generations per year. The model is not stage-specific and this may lead to certain biases in the predicted results; *e*.*g*., it does not account for the photosensitive larval stage under which diapause is induced nor that development is then arrested at the third larval instar. Furthermore, the model does not consider differences in developmental rates between the different instars. Thus, the predicted number of generations has to be interpreted with caution.

Developmental rates of BTM can differ depending on: (1) the geographical origin of the populations (*e*.*g*., showcased by differences in developmental data from Japan [40] and Switzerland [34]); (2) the seasonal generation (*i*.*e*., differences in developmental rates between the post-diapausing and the summer generations [40]) and also of the post-diapausing generation between different populations, at least partly linked to the overwintering life stage [34]; (3) the host plant (*i*.*e*., there are > 15 box tree species or subspecies in the native range of BTM [59]) and observed differences in developmental rates between larval feeding on different host plants [42]; and (4) the varying number of larval instars, which is influenced by rearing temperature and diet [42]. Thus, it is not surprising that independent studies report differing developmental times, temperature thresholds, and degree-days needed for the completion of each generation (*e*.*g*., [34, 40, 60]. While this may have consequences for the accuracy of the present model in certain regions, sensitivity tests for temperature index parameters (DV0-DV4) by Nacambo *et al*. [34] showed that they have little influence on predicted suitability (EI) for BTM in Asia.

The critical daylength for diapause induction in BTM has also been shown to be influenced by several factors: (1) geographical origin of a population (*i*.*e*., different critical daylengths for different populations within Japan [12]); (2) rearing temperature (*i*.*e*., critical daylength decreases with increasing temperature between 15°C (15-16h daylight) and 28°C (12h50 daylight)) but is around 13h30 at 25°C [34, 41–43]; and (3) larval density (*i*.*e*., a high larval density on a given host plant slightly decreases the critical photoperiod for diapause induction compared to isolated larvae on the host plant [11]). For diapause termination, Nacambo *et al*. [34] showed that a cold period of a minimum 45 days at 2°C is needed for a Swiss population of BTM to increase successful development of the adult stage above 50%. Once daily mean temperatures rise in the spring above 10°C, BTM larvae resume their activity and start feeding [44]. However, by rearing a diapausing BTM population from Spain at constant 15 and 25°C, Lopez & Eizaguirre [43] showed that diapause can also terminate spontaneously without a cold period after approximately 100 days, with no differences due to temperature.

Together, variations in temperature-dependent development, diapause induction, and termination all suggest that BTM populations are locally adapted in different regions. If these variances have low plasticity and are genetically inherited, then model predictions for invaded regions may be accurate only if the parameters are adapted to the life-history traits of the invading population. However, if these life-history traits are instead epigenetic, with a high degree of plasticity and dependent on the direct environment of BTM, then rapid adaptations over time may take place in an invading population and the CLIMEX model predictions may only be accurate for certain regions and not for larger areas such as entire continents. Five haplotypes of BTM have been identified in its invaded range across Europe, matching haplotypes of populations in eastern China [22, 61]. Data suggest that there have been several introduction events from China to Europe [22] and this complicates the identification of haplotype-specific life-history traits for BTM. While this may lead to inaccurate estimates of the moth’s phenology in some regions, it seems to have little influence on the predicted potential distribution, as evidenced by the few differences in potential distribution between Nacambo *et al*. [34] and our study. One exception is the predicted unsuitability of tropical regions when the temperature for diapause induction is assumed to be lower than 25°C [34]. Surveys for the occurrence of BTM in wider areas of southern and south-east Asia should be conducted to assess whether this is part of BTM’s native range.

It is important that the northern and southern limits of the predicted distribution be interpreted with caution. Little data are available on the actual distribution of the moth in its native Asian range, so the limits in its area of origin cannot be used alone to make predictions for newly invaded areas. Additionally, cold-induced mortality during diapause and heat-related limitations for development, which are respectively important factors that limit the establishment in northern and southern regions, have not been studied sufficiently for a data-based choice of model parameters under cold and heat stress. Therefore, these parameters were estimated here to the best of our knowledge as described under Material and Methods. Due to these critical data gaps around BTM temperature-dependence, we did not attempt to use our model to predict the potential distribution of BTM under different climate change scenarios. Despite such uncertainties, we know that generally warmer conditions will lead to milder winters and thus provide sufficient degree days for BTM to undergo at least one generation per year, and thus continue to expand northward in regions where it is currently established. As well, in areas where currently there is only one generation per year and negligible damage to *Buxus* spp., a future warming climate will allow for an additional BTM generation per year leading to significantly greater damage in these areas. It is also possible that more frequent extreme weather events predicted with climate change, such as heat waves and droughts, may actually limit the distribution of BTM in localized regions.

It is clear that further biological studies are needed to examine the temperature-dependent effects on BTM to improve our ability to predict its impact and develop effective management. Specifically, BTM’s performance (development and survival) at high temperatures above 30°C and mortality induced by extreme low temperatuers during diapause still need to be investigated. In addition, a better understanding of the moth’s distribution in its native range and potential adaptations and/or genetic differences between populations in different countries within its native range (*e*.*g*., Japan, China, Korea, and Thailand) may help to improve model parameters but also to find appropriate management options, such as specialized and well adapted natural enemies that can be considered for biological control [16].

## Supporting information

S1 File Geographic coordinates for the native and invasive distribution of the box tree moth *Cydalima perspectalis* sourced from the scientific literature and GBIF. (XLSX)

S1 Fig Worldwide potential distribution (yellow to red areas) of the box tree moth, *Cydalima perspectalis*, as predicted by the Ecoclimatic Index of the CLIMEX model developed in the present study. The map was created using CLIMEX and QGIS software.
(TIF)
