# Supplementary figures and images for: Modelling potential distribution of the invasive box tree moth across Asia, Europe, and North America
Source: PLoS One. 2024 Apr 26;19(4):e0302259. doi: 10.1371/journal.pone.0302259 (PMC11051646; doi:10.1371/journal.pone.0302259)

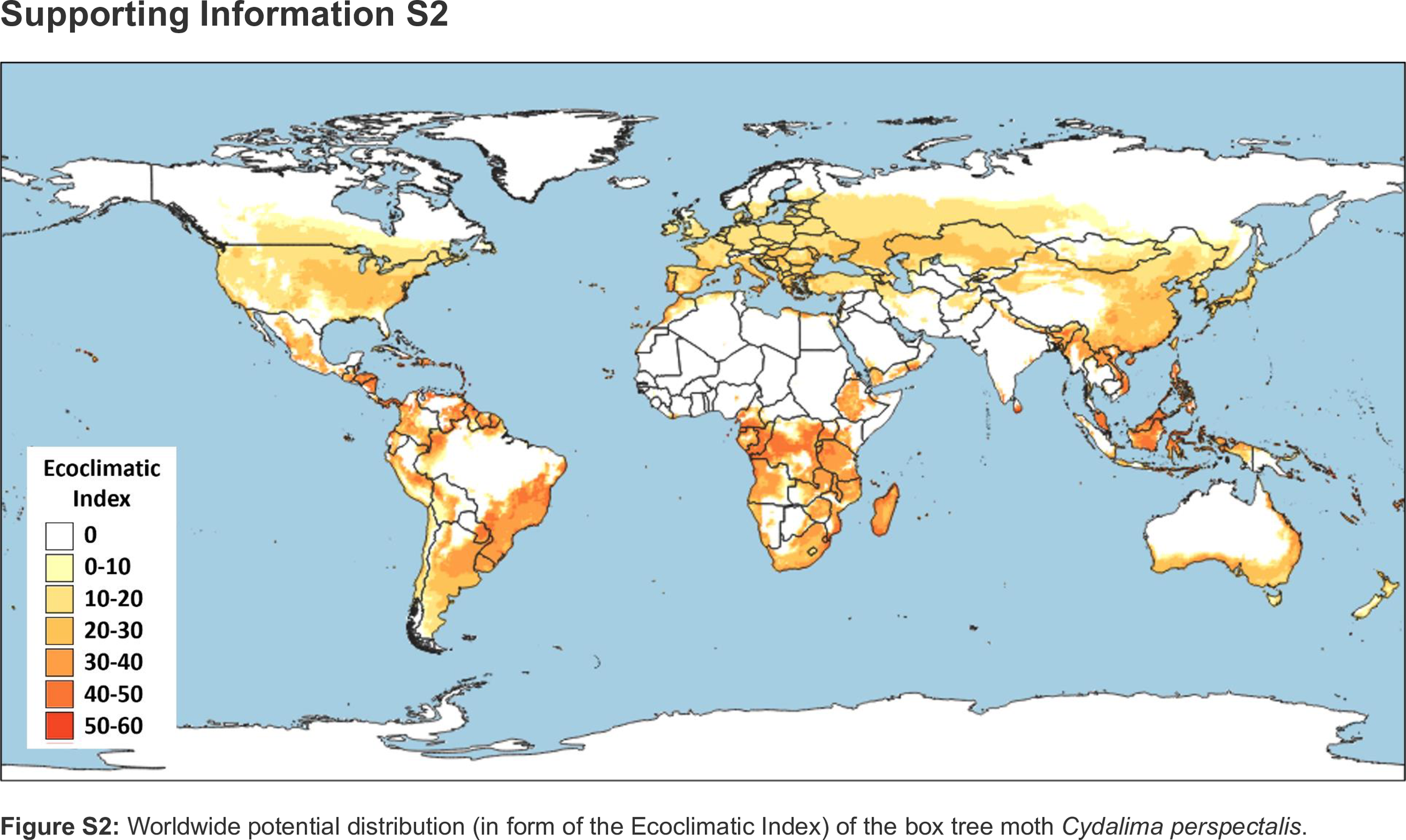

Supplement: S1 Fig — The map was created using CLIMEX and QGIS software. (TIF) [file pone.0302259.s002.tif]
